# Cervical Pott’s disease: case report and review of the literature

**DOI:** 10.11604/pamj.2022.42.299.33951

**Published:** 2022-08-22

**Authors:** Dognon Kossi François de Paule Adjiou, Michèle Yolande Moune, Fresnel Lutèce Ontsi Obame, José Dimbi Makoso, Nourou Dine Adeniran Bankole, Mustapha Hemama, Nizare El Fatemi, Mouley Rachid El Maaqili

**Affiliations:** 1 Faculty of Medicine and Pharmacy, Mohamed V University of Rabat, Rabat, Morocco,; 2 Department of Neurosurgery, Ibn Sina Hospital, Rabat, Morocco

**Keywords:** Tuberculosis, spinal, management, case report

## Abstract

Tuberculosis is a major public health problem in the world. Spinal tuberculosis (Pott disease) is a frequently encountered extrapulmonary form of the disease. Cervical spinal tuberculosis is relatively rare. We report the case of a 66-year-old patient admitted for cervical Pott's disease managed surgically and the positive outcome. A patient with a history of pulmonary tuberculosis present 3 months ago persistent neck pain with tingling and heaviness in both upper limbs. The neurological examination was normal without any sensory or motor deficit. Spinal cord magnetic resonance imaging (MRI) showed a lesion centered on the vertebral body of C4 with spinal cord compression and epiduritis without signs of spinal cord injury. The patient underwent a corpectomy of C3 and C4 with an iliac graft and anterior cervical plate. The anatomopathological examination revealed a Pott disease. He was therefore put on antituberculous chemotherapy for 12 months. Three months later the neck pain and tingling disappeared in the upper limbs. Cervical Pott's disease is relatively rare. Surgical management is indicated in the case of spinal instability or spinal cord compression.

## Introduction

Tuberculosis is a major public health problem in the world. Spinal tuberculosis (TB) is the most common musculoskeletal manifestation, affecting about 1 to 2% of all cases of TB [1]. Cervical spinal tuberculosis (Pott's disease) is relatively rare, and its incidence varies from 2% to 12% [2]. The treatment approaches to cervical spine tuberculosis fluctuated between conservative therapy and surgery [3]. We report a case of cervical Pott's disease managed surgically and the positive outcome in the light of previous literature.

## Patient and observation

**Clinical presentation:** a 66-year-old patient with a history of pulmonary tuberculosis treated in 1997, declared cured, and high blood pressure present three months ago persistent neck pain with tingling and heaviness in both upper limbs. At the neurological examination, the muscular testing was normal and no sensory deficit. The American Spinal Injury Association (ASIA) score was graduated E.

**Figure 1:**
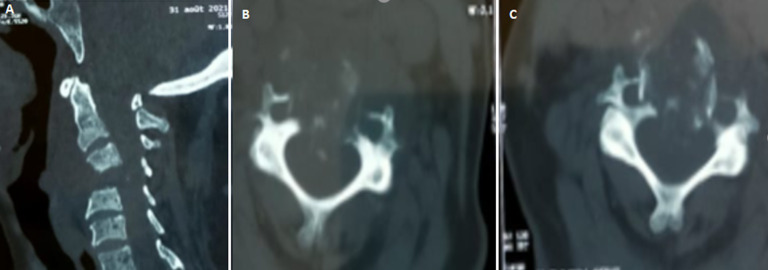
(A,B,C) cervical spine CT, lytic process centered on C4 with posterior wall recession

**Figure 2:**
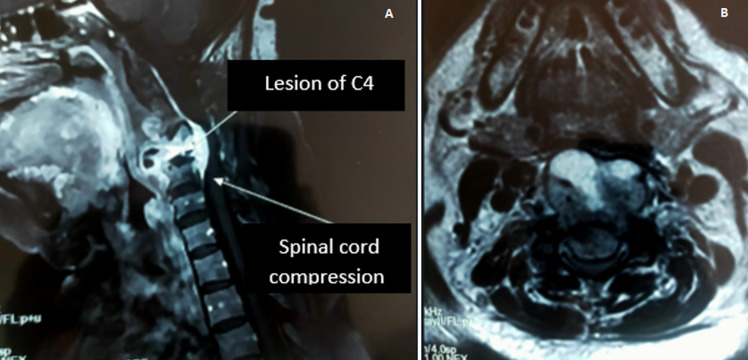
(A,B) spinal cord MRI, cervical lytic lesion on the vertebral body of C4 with spinal cord compression and epiduritis

**Diagnosis assessment:** cervical spine computed tomography (CT) showed a lytic process centered on C4 with posterior wall recession and anterior epiduritis (Figure 1). Spinal cord magnetic resonance imaging (MRI) showed a cervical lytic lesion centered on the vertebral body of C4 with spinal cord compression and epiduritis without signs of spinal cord injury (Figure 2).

**Figure 3:**
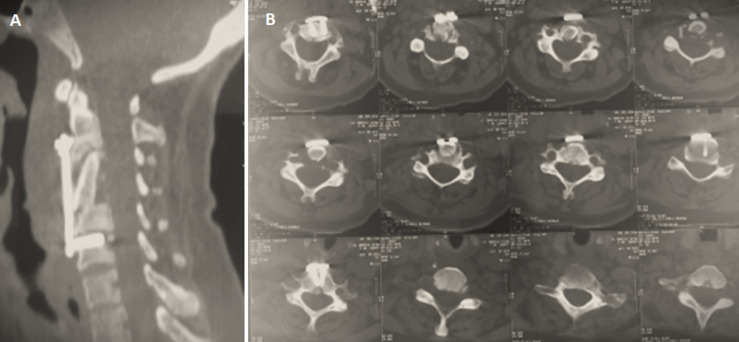
(A,B) corpectomy of C3 and C4 with iliac graft and anterior cervical plate

**Management:** the patient underwent corpectomy of C3 and C4 with iliac graft and anterior cervical plate (Figure 3). Then a reinforced neck brace was applied 2 weeks after the surgery to enable consolidation. The anatomopathological examination revealed a Pott disease. The patient was put on antituberculosis chemotherapy treatment for 12 months: 2 months of Rifampicin - Isoniazid - Pyrazinamid - Ethambutol (RHZE) followed by 10 months of Rifampicin-Isoniazid (RH).

**Outcomes:** at discharge, 2 weeks after surgery, the patient presented the same neurological state as at admission.

**Follow up:** the patient was seen in outpatient three months later. No complaint was noted, and neurological examination was normal.

**Declaration of patient consent:** the authors certify that they have obtained all appropriate patient consent.

## Discussion

We report the case of a 66-year-old patient with a history of treated pulmonary tuberculosis in 1997, admitted for cervical pains and heaviness of the upper limbs, ASIA E, and in whom the explorations came back in favour of a cervical Pott's disease that we managed surgically by an anterior approach. The management of stable cervical spine Pott's disease was previously controversial. The surgical treatment of cervical spine Pott’s disease is practiced worldwide. However, its indications are well described in literature [4] and they are based on results of clinical and radiologic investigations.

The clinical presentation of cervical spine Pott’s disease is not specific. The average time between the onset of symptoms and diagnosis is highly variable from one to several months (Table 1, Table 1 suite). The clinical signs can range from isolated neck pain to a sensitivo-motor deficit whoever the main symptoms at onset are neck pain and restricted neck movement. The smaller canal dimension, proximity to vertebral artery and other vital structures, unique facetal architecture, higher mobility and lordotic alignment make the cervical spine vulnerable to greater neuro-deterioration, instability, and progressive mal-alignment [5]. Our patient presented persistent neck pain with tingling and heaviness in both upper limbs. The average time from symptom onset to diagnosis was 3 months.

**Table 1:** review of literature on cervical spine Pott disease

Authors	Cases	Level	Mean age	Sex	clinic	ASIA	Follow up	Disease duration	Surgical approach	Outcome
Xin Hua *et al*.	87 cases; 78 surgery; 9 conservative	C0-C7; 1 level 68; 2 level 2; multiple 8	Mean age 36.9 ± 1.6 y	M 43; F 35	Neck pain 78; stiffness/restricted neck range motion 78; dysphagia/dyspnea 20; loss of appetite 67; low grade fever 35; progressive torticollis 25; weight loss 50	ASIA pre (A) A0 B6 C19 D11 E 1; ASIA post (A) A0 B0 C0 D3 E 34; ASIA pre (AP) A0 B8 C12 D 9 E 0; ASIA post (AP) A0 B0 C0 D1 E28; ASIA pre (P) A0 B2 C7 D 3 E3; ASIA post (P) A0 B 0 C0 D1 E11	Follow up 41.5 ± 7.2 months	Disease duration 4.5 ± 1.1 months	A 37; AP29; P12	Improved 73; unchanged 4
Maolin He *et al*.	25 cases	C3-C7; 1 level 3; 2 level 18; multiple 4	39y	M 18; F 7	Neck pain 25; neck stiffness 25; spastic quadriparesis 20; cervical radiculopathy 9; constitutional symptom 6; sphincteric disturbance 2	ASIA pre op A0 B3 C5 D12 E5; ASIA post op A0 B0 C0 D5 E20	37.5 months	NR	A	Improved 18; unchanged 2
Wence Wu *et al*.	58 cases; 17 surgery; 35 conservative; 6 lost to follow-up	C4-C7; 1 level 5; 2 level 12	45.7 ± 16.8 y	M 9; F 8	Clinical symptoms percentage NR	Frankel pre op A2 B3 C3 D6 E3 Frankel Post op A0 B1 C3 D4 E9	45.5 ± 12.9 months	NR	A	Improved 14
Torphong Bunmaprasert *et al*.	18 cases; 16 surgery; 2 conservative	C0-C7	51.4 y	M 13; F 5	Neck pain 18; progressive quadriparesis 12; spastic gait 7; hand clumsiness 8	Nurick preoperative grade 0 :1; grade II: 1; grade III : 3; grade IV : 9; grade V : 4; no improvement in conservative management	12.8 months	5.5 months	A 13; P 3	Improved 14

NR: no record; M: male; F: female

**Table 1(suite):** review of literature on cervical spine Pott disease

Authors	Cases	Level	Mean age	Sex	Clinic	ASIA	Follow up	Disease duration	Surgical approach	Outcome
Shuai Xing *et al*.	11 cases	C1-C2	40.4 ± 9.5 y	M 7; F 4	NR	JOA pre op 8.4 ± 1.3; JOA post op 15.0 ± 1.3	39 months	17.3 ± 4.5 months	AP	Improved 9; unchanged 2
Ningfang Mao *et al*.	21 cases	C3-C7 1 level 18; 2 level 3	37 ± 8.9 y	M 13; F 8	Cervical pain 21; low grade fever 19; night sweats 19; spinal neurological impairement 19; emaciation 16; fatique 12; sphincter dysfunction 4	Frankel pre op A0 B2 C6 D10 E3; Frankel post op A0 B0 C1 D2 E18	72.4 months	8 ± 2.8 months	A	Improved 18; unchanged 3
Elsawaf *et al*.	29 cases; 16 surgery; 13 conservative	NR	23.7 y	M 22; F 7	NR	NR	14 months	NR	A 16	Neurological improvement in both approaches more obvious in surgical group
Koptan *et al*.	30 cases	NR	44.5 y	M 14; F 16	NR	NR	66 months	NR	A	NR
Raja *et al*.	44 cases	1 level 42 Multiple 2	NR	M 20; F 24	NR	NR	12 months	NR	A	All patients had good neurologic recovery
Wang *et al*.	66 cases;26 surgery; 40 conservative	NR	51.3 y	NR	NR	NR	26.5 months	NR	A 22; AP 4	All patients had good neurologic recovery at 4 months follow up

NR: no record; M: male; F: female; JOA: Japanese orthopaedic association

Because of the high risk of neurological deficits due to epidural compression of the cervical spine or spinal instability, the diagnosis should be made at the first symptoms and investigations should be done accordingly. Magnetic resonance imaging is considered the investigation of choice in spinal infection because it has high sensitivity and satisfactory specificity. The advantages of CT over MRI are that CT offer more reliable detection of calcified foci and they also provide guidance on the need for interventional procedures. Anterior approach is the gold standard in surgical management because lesions are located predominantly on the anterior column [2]. Performing anterior debridement and bone grafting fusion with instrumentation, has the advantages of direct access to the focus of the disease, bony union, and stabilization of the spine. Anti-TB treatments are systematic as soon as the bacteriological examination confirms the presence of mycobacterium tuberculosis.

Surgery and conservative management are treatment options of spinal TB. Surgical procedure should be considered in patients with extensive spine involvement, severe deformity, vertebral body collapse, prevertebral cervical abscess, advanced neurological involvement, and any sign of progressive recovery despite of conservative therapy [6]. Our patient underwent a corpectomy of C3 and C4 with iliac graft and anterior cervical plate. Then a reinforced neck brace was applied for 3 months. The evolution is often favorable when patients are managed as soon as the first symptoms appear.

The anterior approach is the most used. Most patients showed an improvement of the neurological status in case of preoperative neurological deficit. In our case, the patient benefited from an anterior approach with improvement of neurological status 3 months after surgery. Table 1 and Table 1 (suite) summarizes the data from the literature review [2,7-15].

## Conclusion

Tuberculosis is an empirical but still prevalent infectious disease. It is a real public health problem. Cervical vertebral localizations, although rare, can always occur in patients treated for pulmonary tuberculosis. The management is based on anti-tuberculosis treatments and surgery by posterior, anterior or combined approaches is indicated on a case-by-case basis if spinal instability or spinal cord compression.

**Disclosures:** the authors did not receive any funding for the preparation of this case report. This article is an original work that is not being considered or reviewed by any other publication and has not been published elsewhere in the same or a similar form. All authors of the manuscript have read and agreed to its content and are accountable for all aspects of the accuracy and integrity of the manuscript.
